# Ischemia-modified albumin in children with clinically suspected acute myocarditis: diagnostic performance and incremental value beyond conventional biomarkers

**DOI:** 10.3389/fped.2026.1844353

**Published:** 2026-06-15

**Authors:** Ceren Yapar Gümüş, Emine Yurdakul Ertürk, Yeliz Kaşko Arıcı, Ecem İpek Altınok, Tülin Bayrak, Ahmet Bayrak, Taner Kasar

**Affiliations:** 1 Faculty of Medicine, Department of Pediatrics, Ordu University, Ordu, Türkiye; 2 Faculty of Medicine, Department of Biostatistics and Medical Informatics, Ordu University, Ordu, Türkiye; 3 Faculty of Medicine, Department of Medical Biochemistry, Ordu University, Ordu, Türkiye; 4 Faculty of Medicine, Department of Pediatric Cardiology, Ordu University, Ordu, Türkiye

**Keywords:** biomarkers, child, ischemia-modified albumin, myocarditis, oxidative stress, ROC curve

## Abstract

**Background:**

Pediatric acute myocarditis is challenging because presentations range from chest pain to cardiogenic shock, and no single biomarker reflects its inflammatory, ischemic, and hemodynamic components. Ischemia-modified albumin (IMA), a marker of oxidative stress-related alteration in albumin measured by the albumin cobalt-binding assay, has been investigated in ischemic and inflammatory conditions, but its role in pediatric myocarditis remains unclear.

**Methods:**

In this prospective, single-center case-control study, 47 children with clinically suspected acute myocarditis and 47 age- and sex-matched healthy controls were evaluated between 1 July 2023 and 1 July 2025. Clinically suspected acute myocarditis was defined according to pediatric guidance as at least one compatible clinical feature together with at least one supportive biomarker, electrocardiographic, or echocardiographic finding after exclusion of alternative diagnoses. IMA levels were measured using the albumin cobalt-binding method and expressed as absorbance units (ABSU). The statistical analysis included between-group comparisons, receiver operating characteristic (ROC) analyses, incremental discrimination analyses, and exploratory clinical severity analyses.

**Results:**

IMA concentrations were significantly higher in the myocarditis group than in controls [0.57 (0.55–0.63) vs. 0.55 (0.49–0.59) ABSU, *p* = 0.021], with a Hodges–Lehmann median difference of 0.04 ABSU (95% CI, 0.01–0.07). However, the diagnostic performance of IMA alone was modest (AUC, 0.64; 95% CI, 0.52–0.75). At the optimal cutoff of 0.53 ABSU, sensitivity was 89.36% and specificity was 38.30%. Conventional biomarkers showed stronger discrimination, including troponin I (AUC, 0.96), troponin T (AUC, 0.93), NT-proBNP (AUC, 0.88), CRP (AUC, 0.85), and CK-MB (AUC, 0.83). A base model including CK-MB, NT-proBNP, and CRP yielded an AUC of 0.92; adding IMA increased the AUC marginally to 0.93 (delta AUC, 0.01; *p* = 0.153). All children in the clinically suspected myocarditis group were symptomatic at presentation, and exploratory analyses showed no significant association between IMA and selected electrocardiographic or echocardiographic abnormalities or length of hospital stay.

**Conclusion:**

IMA is elevated in children with clinically suspected acute myocarditis, but its standalone diagnostic performance is limited and its added value beyond conventional biomarkers appears small. IMA may be considered an adjunctive rather than a primary diagnostic biomarker, and any putative triage role in ultra-early presentations remains hypothesis-generating.

## 1 Introduction

Acute myocarditis is an inflammatory disease of the myocardium characterized by myocardial edema, cellular injury, and, in some cases, myocyte necrosis (1–4). In children, clinical presentation is highly variable, ranging from nonspecific symptoms such as fever, abdominal pain, vomiting, palpitations, dyspnea, and chest pain to severe heart failure, ventricular arrhythmias, marked systolic dysfunction, or sudden cardiac death (1–5). Although many children recover fully, a clinically important subgroup develops persistent ventricular dysfunction, dilated cardiomyopathy, or requires advanced heart failure therapies (1, 3, 4). The burden of pediatric myocarditis is likely underestimated because mild or transient cases may remain unrecognized (3, 6, 7). Nevertheless, available epidemiologic data indicate that it is uncommon but clinically significant. A nationwide study from Finland reported an incidence of 1.95 per 100,000 person-years, whereas a large hospitalization-based analysis from the United States estimated an incidence of approximately 0.8 per 100,000 children and showed increasing hospitalization rates over time (6, 7). Its clinical importance is further underscored by autopsy-based evidence identifying myocarditis as a cause of sudden death in young individuals, including athletes (5). Diagnosis usually relies on the integration of clinical findings, electrocardiography, echocardiography, cardiac biomarkers, and, when available, cardiac magnetic resonance imaging (1, 2, 8). Troponins and natriuretic peptides remain central because they reflect myocardial injury and ventricular stress more directly than nonspecific inflammatory markers, yet no single test is definitive in all children (1, 2). Endomyocardial biopsy is invasive and selectively used, and cardiac magnetic resonance may not be immediately available at every presentation (1, 2, 8). These limitations have sustained interest in adjunctive biomarkers that may reflect complementary pathobiological pathways. Ischemia-modified albumin (IMA) is formed when oxidative stress, hypoxia, acidosis, and free radical-mediated injury alter the N-terminal metal-binding region of albumin, reducing its affinity for cobalt (9–11). Although initially investigated as a marker of myocardial ischemia, IMA is now regarded as a broader indicator of oxidative and ischemia-related biochemical stress rather than a myocardium-specific biomarker (9–12). This may be relevant in myocarditis, where inflammatory and oxidative mechanisms coexist, but pediatric evidence remains scarce. Increased IMA levels have been reported in children with acute rheumatic fever and in adult studies of acute myocardial injury and myocarditis-related conditions (12–14). However, pediatric studies specifically evaluating IMA in acute myocarditis are extremely limited. Therefore, we aimed to assess serum IMA levels in children with clinically suspected acute myocarditis, evaluate its diagnostic performance, and determine whether it provides incremental discriminatory value beyond conventional biomarkers.

## 2 Materials and methods

### 2.1 Study design and setting

This prospective, single-center case-control study was conducted at Ordu University Women's and Children's Hospital between 1 July 2023 and 1 July 2025. Participants were recruited through the pediatric cardiology outpatient clinic, general pediatrics outpatient clinic, and pediatric emergency department. The study was designed to evaluate serum ischemia-modified albumin levels in children with clinically suspected acute myocarditis and to compare their diagnostic performance with that of conventional biomarkers. The final study population consisted of 47 children in the myocarditis group and 47 healthy controls.

### 2.2 Participants and case definition

Children aged 1 month to 18 years were eligible for inclusion if they were evaluated by a pediatric cardiologist and fulfilled the predefined criteria for clinically suspected acute myocarditis. Case classification was based on the 2021 American Heart Association scientific statement on myocarditis in children and the 2024 American College of Cardiology Expert Consensus Decision Pathway, published in 2025, on diagnostic and management strategies for myocarditis (1, 2). In the present study, clinically suspected acute myocarditis was defined as the presence of at least one compatible clinical feature together with at least one supportive objective finding, after exclusion of alternative diagnoses. Compatible clinical features included chest pain, palpitations, dyspnea, and exercise intolerance. Supportive findings included one or more of the following: elevation of cardiac biomarkers, including troponin I (TnI), troponin T (TnT), creatine kinase-myocardial band (CK-MB), or N-terminal pro-B-type natriuretic peptide (NT-proBNP); electrocardiographic (ECG) abnormalities such as ST-T changes, T-wave inversion, arrhythmia, or conduction disturbance; or echocardiographic abnormalities including reduced left ventricular ejection fraction, regional wall-motion abnormality, ventricular dilatation, or pericardial effusion. Patients were included only after other clinically plausible explanations had been excluded.

### 2.3 Inclusion and exclusion criteria

The inclusion criteria were age between 1 month and 18 years and fulfillment of the study definition for clinically suspected acute myocarditis. The exclusion criteria were congenital heart disease, age younger than 1 month, concomitant lower respiratory tract infection, known chronic disease, regular medication use, refusal or withdrawal of participation, and establishment of an alternative diagnosis based on clinical and laboratory inconsistency.

### 2.4 Control group

**Figure 1:**
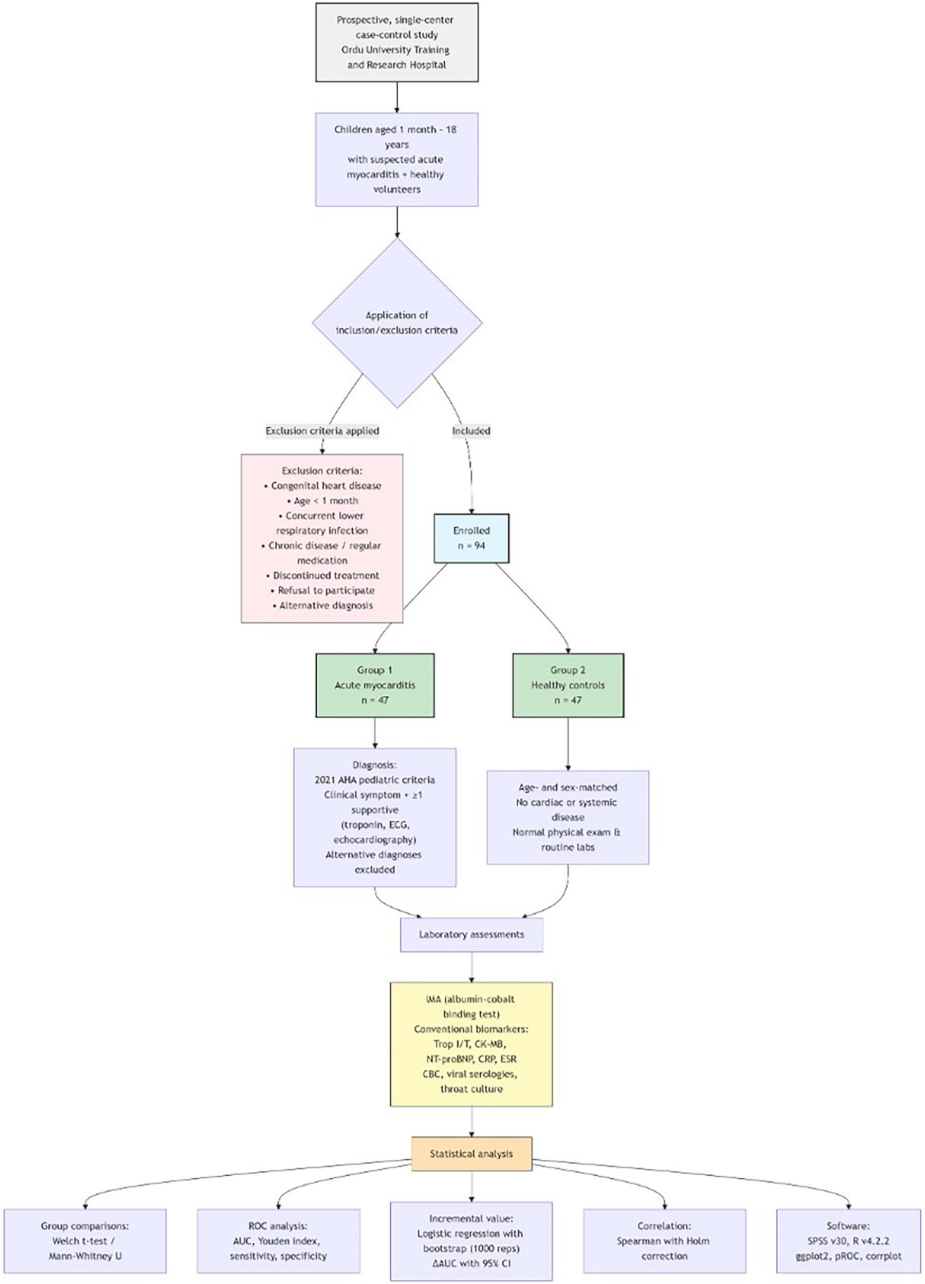
Study design and participant grouping of the single-center case–control study. This schematic summarizes the prospective single-center case–control workflow, including prespecified inclusion/exclusion criteria, allocation into clinically suspected acute myocarditis (*n* = 47) and age-/sex-matched healthy controls (*n* = 47), laboratory measurements (IMA and conventional biomarkers), and the planned statistical analyses (group comparisons, ROC analysis, incremental discrimination modeling, clinical severity analyses, and correlation analysis). Numbers represent the final analyzed sample.

The control group consisted of healthy children without a known history of cardiac or systemic disease. Controls were selected to be age- and sex-matched to the myocarditis group. Written informed consent was obtained from the parents or legal guardians of all control participants before enrollment. The study flow, participant selection process, exclusions, and final group allocation are summarized in Figure 1.

### 2.5 Clinical, electrocardiographic, echocardiographic and laboratory assessment

For each participant, demographic characteristics, presenting symptoms, physical examination findings, electrocardiographic findings, echocardiographic findings, and laboratory measurements obtained at presentation were recorded. The laboratory variables evaluated in the study included complete blood count, C-reactive protein (CRP), erythrocyte sedimentation rate (ESR), TnI, TnT, CK-MB, and NT-proBNP. These values represented the first available measurements obtained at admission or initial evaluation. Serum samples collected for ischemia-modified albumin analysis were separated and stored at −80 °C until batch analysis in the biochemistry research laboratory.

### 2.6 Measurement of ischemia-modified albumin

Serum ischemia-modified albumin levels were measured in the biochemistry research laboratory using the colorimetric albumin cobalt-binding assay described by Bar-Or et al. (15). Briefly, 200 μL of serum was mixed with 50 μL of 0.1% cobalt chloride solution and incubated for 10 min in the dark to allow cobalt–albumin binding. Subsequently, 50 μL of dithiothreitol solution at a concentration of 1.5 mg/mL was added, and after 2 min, 1.0 mL of 0.9% sodium chloride was added to terminate the reaction. Absorbance was measured at 470 nm using a spectrophotometer, and the results were expressed as absorbance units (ABSU). Serum samples were stored at −80 °C until batch analysis, with an approximate storage duration of up to six months before IMA measurement. To minimize potential degradation effects, repeated freeze–thaw cycles were strictly avoided, and all samples underwent only a single freeze–thaw cycle prior to analysis. All measurements were performed in duplicate, and the mean of the two readings was used for statistical analysis. In addition, samples showing evidence of hemolysis, lipemia, or icterus were excluded to minimize potential analytical interference and to ensure assay reliability. All samples were analyzed in the same analytical run to minimize between-run variability. However, formal study-specific intra-assay and inter-assay coefficients of variation and separate calibration or quality-control records were not available for retrospective reporting.

### 2.7 Study outcomes

The primary outcome of the study was the difference in serum ischemia-modified albumin levels between children with clinically suspected acute myocarditis and healthy controls. Secondary outcomes were the diagnostic performance of ischemia-modified albumin assessed by receiver operating characteristic curve (ROC) analysis, determination of the optimal cut-off value, and evaluation of its incremental discriminatory contribution beyond conventional biomarkers. Exploratory analyses included associations between IMA and presenting clinical features, selected electrocardiographic and echocardiographic abnormalities, length of hospital stay, and other inflammatory and cardiac biomarkers within the clinically suspected myocarditis group.

### 2.8 Statistical analysis

Statistical analyses were conducted using IBM SPSS Statistics v30 (IBM Corp., Armonk, NY, USA). Continuous variables were inspected for distributional assumptions using graphical methods (histograms and Q–Q plots) and the Shapiro–Wilk test. Variables consistent with approximate normality were summarized as mean ± standard deviation (SD) and compared between groups using Welch's *t*-test. Non-normally distributed variables were summarized as median [Q1–Q3] and compared using the Mann–Whitney *U*-test. Categorical variables, when applicable, were summarized as counts (percentages) and compared using the Pearson *χ*^2^-test or Fisher's exact test as appropriate. All tests were two-sided and *p* < 0.05 was considered statistically significant.

### 2.9 Handling values below the limit of detection (LOD)

For biomarkers reported as below the limit of detection (<LOD), values were imputed as LOD/2 for primary analyses to allow continuity of numeric modeling, while a binary indicator (<LOD: yes/no) was retained for transparency and sensitivity analyses. Given the expected floor effect for troponins in healthy controls, interpretation of diagnostic performance prioritized ROC-based metrics alongside distributional summaries.

### 2.10 Diagnostic performance and ROC analysis

Diagnostic performance of each biomarker for discriminating myocarditis cases from controls was evaluated using receiver operating characteristic (ROC) curve analysis. The area under the ROC curve (AUC) was reported with 95% confidence intervals. AUC 95% confidence intervals were obtained using bootstrap resampling. Optimal cut-off values were determined using the Youden index, and corresponding sensitivity and specificity were reported. To evaluate the incremental discrimination contributed by IMA beyond conventional biomarkers, multivariate binary logistic regression models were constructed using clinically relevant biomarkers (log10-transformed CK-MB, NT-proBNP, and CRP) and then refit with IMA added to the model. Discrimination was quantified using model-predicted probabilities and ROC curves; the uncertainty of *Δ*AUC between nested models was assessed using bootstrap resampling. As an exploratory analysis, pairwise differences in AUC between IMA and each conventional biomarker were estimated by nonparametric bootstrap resampling and summarized with 95% confidence intervals.

As exploratory analyses within the clinically suspected myocarditis group, IMA levels were compared according to abnormal ECG findings, arrhythmia/rhythm disturbance, any echocardiographic abnormality, left ventricular systolic dysfunction, and pericardial effusion using the Mann–Whitney *U*-test, and the association between IMA and length of hospital stay was assessed using Spearman's rank correlation coefficient. Because several conventional biomarkers contributed to the pragmatic case definition, ROC analyses for those biomarkers were interpreted cautiously as exploratory comparisons subject to potential incorporation bias.

### 2.11 Correlation analysis

Associations between biomarkers within the myocarditis group were examined using Spearman's rank correlation coefficient (*ρ*). For correlation matrices, two-sided *p*-values were computed and, where multiple comparisons were performed, *p*-values were adjusted using a Holm procedure to control the family-wise error rate. Correlation results were presented as (*ρ*, *p*) in the corresponding heatmap.

### 2.12 Data visualization

Figures were generated in R 4.5.0 using RStudio 2026.01.1 + 403 (Windows) and Quarto 1.8.25.

### 2.13 Ethics

The study protocol was approved by the Ordu University Clinical Research Ethics Committee (Decision No. 2023/163) and was conducted in accordance with the principles of the Declaration of Helsinki. Written informed consent was obtained from the parents or legal guardians of all participants before enrollment.

## 3 Results

**Figure 2:**
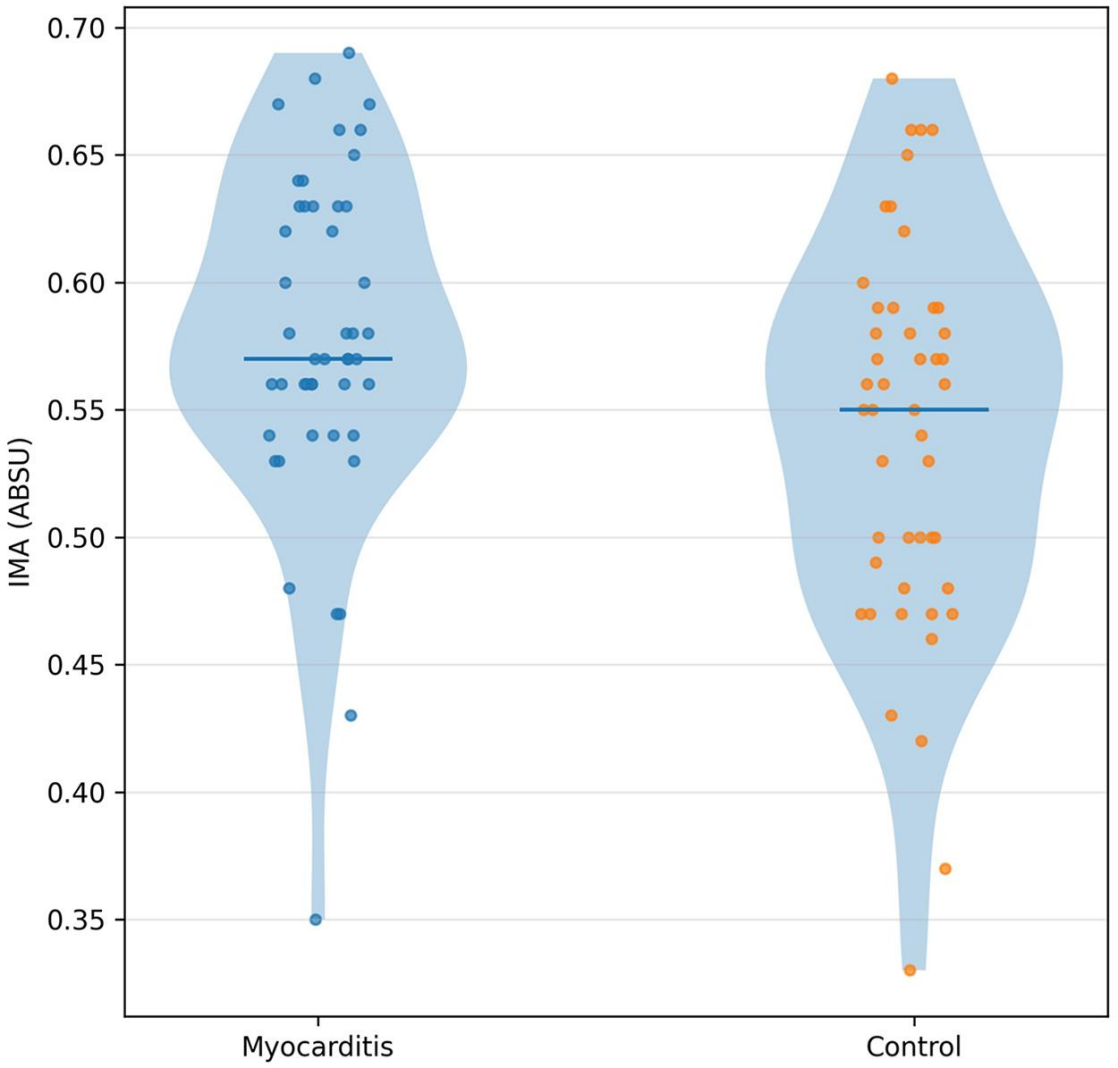
Violin plot of IMA (clinically suspected acute myocarditis vs. Control). Violin plots summarize the distribution of ischemia-modified albumin (IMA, ABSU) in each group; overlaid points represent individual participants (jittered to reduce overlap). The central tendency shown corresponds to the median distributional location; between-group comparison was performed using the Mann–Whitney *U*-test (Table 1).

Baseline characteristics and laboratory findings are summarized in Table 1. Age distribution was comparable between groups. Inflammatory markers and cardiac biomarkers were markedly higher in the clinically suspected myocarditis group, including CRP, ESR, troponins, CK-MB, and NT-proBNP (all *p* < 0.001). IMA was modestly but significantly higher in clinically suspected myocarditis than in controls [0.57 (0.55–0.63) vs. 0.55 (0.49–0.59), *p* = 0.021]. The distribution of IMA values in the two groups is illustrated in Figure 2. The Hodges–Lehmann median difference for IMA was 0.04 ABSU (95% CI 0.01–0.07). No meaningful between-group differences were observed for age, hemoglobin, platelet count, or lymphocyte count, whereas inflammatory and cardiac biomarkers were consistently higher in the myocarditis group.

**Table 1:** Baseline clinical and laboratory characteristics of children with clinically suspected acute myocarditis and healthy controls.

**Variable**	**Clinically suspected acute myocarditis (*n* = 47)**	**Control (*n* = 47)**	**P**
Age (years)	13.00 [10.00–16.00]	13.00 [10.00–16.00]	0.979ᵇ
Hemoglobin (g/dL)	12.82 ± 1.97	13.52 ± 1.41	0.052ᵃ
White blood cells (/mm^3^)	8,320.00 [7,225.00–11,180.00]	6,700.00 [5,625.00–8,875.00]	**0.003** ᵇ
Platelets (/mm^3^)	289,297.87 ± 110,352.97	319,978.72 ± 81,887.11	0.130ᵃ
Neutrophils (/mm^3^)	5,110.00 [3,805.00–6,860.00]	3,510.00 [2,935.00–4,910.00]	**0.002** ᵇ
Lymphocytes (/mm^3^)	2,250.00 [1,965.00–2,960.00]	2,180.00 [1,895.00–2,910.00]	0.868ᵇ
CRP (mg/L)	13.00 [1.71–34.05]	0.57 [0.15–1.60]	**<0.001** ᵇ
Troponin I (ng/mL)	1.33 [0.49–5.91]	0.05 [0.05–0.05]	**<0.001** ᵇ
Troponin T (pg/mL)	173.00 [20.80–346.00]	1.50 [1.50–4.03]	**<0.001** ᵇ
CK-MB (ng/mL)	5.99 [3.01–20.42]	1.82 [1.29–2.52]	**<0.001** ᵇ
ESR (mm/h)	30.00 [17.00–34.50]	13.00 [8.50–22.00]	**<0.001** ᵇ
NT-proBNP (ng/L)	208.00 [68.10–1,005.00]	35.90 [13.00–51.00]	**<0.001** ᵇ
IMA (ABSU)	0.57 [0.55–0.63]	0.55 [0.49–0.59]	**0.021** ᵇ

Data are presented as mean ± SD or median [Q1–Q3].

ᵃ Welch *t*-test.

ᵇ Mann–Whitney *U*-test.

Bold *p*-values indicate statistical significance at *p* < 0.05.

*Clinical characteristics of the clinically suspected myocarditis group*

Clinical characteristics of the clinically suspected myocarditis group are summarized in Table 2. All 47 children were symptomatic at presentation; no asymptomatic cases were identified in the structured dataset. Chest pain was present in all patients (47/47, 100.0%), whereas dyspnea was recorded in 11/47 (23.4%) and fever and cough in 6/47 (12.8%) each. ECG findings were normal in 3/47 (6.4%) and abnormal in 44/47 (93.6%), most commonly with ST-T abnormalities (40/47, 85.1%). Echocardiography was normal in 20/47 (42.6%) and abnormal in 27/47 (57.4%); left ventricular systolic dysfunction and pericardial effusion were each present in 9/47 (19.1%), and left ventricular dilatation in 4/47 (8.5%). Median length of hospital stay was 5.00 [3.00–8.50] days.

**Table 2:** Clinical, electrocardiographic, echocardiographic, and hospitalization characteristics of children with clinically suspected acute myocarditis.

**Characteristic**	***n* (%) or summary**
Symptomatic at presentation	47 (100.0)
Asymptomatic at presentation	0 (0.0)
Chest pain	47 (100.0)
Dyspnea	11 (23.4)
Fever	6 (12.8)
Cough	6 (12.8)
Left arm numbness	5 (10.6)
Abdominal pain	5 (10.6)
Vomiting	4 (8.5)
Fatigue	4 (8.5)
Palpitations	4 (8.5)
Syncope	3 (6.4)
Nausea	2 (4.3)
Irritability	1 (2.1)
ECG normal	3 (6.4)
Any ECG abnormality	44 (93.6)
ST-T abnormality	40 (85.1)
Arrhythmia/rhythm disturbance	8 (17.0)
Echocardiography normal	20 (42.6)
Any echocardiographic abnormality	27 (57.4)
Left ventricular systolic dysfunction	9 (19.1)
Pericardial effusion	9 (19.1)
Left ventricular dilatation	4 (8.5)
Length of hospital stay, days	5.00 [3.00–8.50]

Symptom categories and several electrocardiographic and echocardiographic findings were non-mutually exclusive; therefore, percentages may sum to more than 100%.

*Associations between IMA and selected clinical severity indicators*

Exploratory analyses of IMA according to selected indicators of presentation severity are summarized in Table 3. IMA was not significantly associated with abnormal ECG findings (*p* = 0.347), arrhythmia/rhythm disturbance (*p* = 0.186), any echocardiographic abnormality (*p* = 0.545), left ventricular systolic dysfunction (*p* = 0.254), or pericardial effusion (*p* = 0.755). Length of hospital stay likewise showed no significant correlation with IMA (Spearman *ρ* = −0.193, *p* = 0.193; *n* = 47).

**Table 3:** IMA according to selected clinical severity indicators in children with clinically suspected acute myocarditis.

Indicator	Positive subgroup, *n*	IMA in positive subgroup (ABSU)	Reference subgroup, *n*	IMA in reference subgroup (ABSU)	*p*
Any ECG abnormality	44	0.57 [0.56–0.63]	3	0.54 [0.54–0.58]	0.347
Arrhythmia/rhythm disturbance	8	0.56 [0.54–0.57]	39	0.58 [0.56–0.63]	0.186
Any echocardiographic abnormality	27	0.57 [0.54–0.62]	20	0.58 [0.56–0.63]	0.545
Left ventricular systolic dysfunction	9	0.57 [0.53–0.58]	38	0.57 [0.56–0.63]	0.254
Pericardial effusion	9	0.58 [0.54–0.66]	38	0.57 [0.56–0.63]	0.755

Data are presented as median [Q1–Q3]; *p*-values were obtained using the Mann–Whitney *U*-test. For length of hospital stay, Spearman *ρ* = −0.193 and *p* = 0.193 (*n* = 47).

### 3.1 ROC analysis

**Figure 3:**
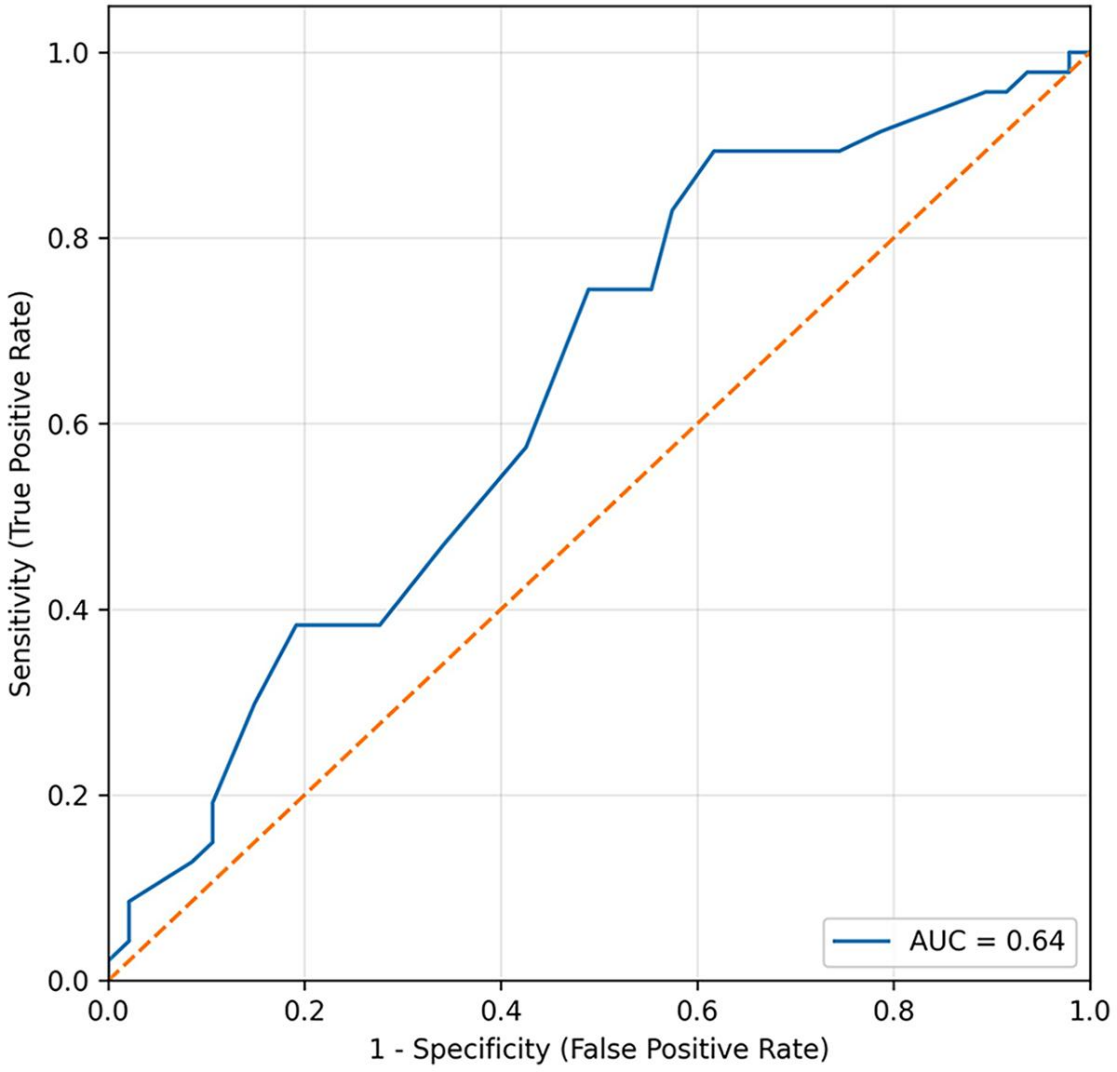
ROC curve for IMA. Receiver operating characteristic (ROC) curve showing the ability of IMA to discriminate clinically suspected acute myocarditis cases from controls. The AUC with 95% confidence interval was obtained using bootstrap resampling, and the optimal cut-off was selected using the Youden index. The corresponding sensitivity and specificity at the Youden-optimal cut-off are reported in Table 4.

ROC performance is summarized in Table 4, and the ROC curve for IMA is shown in Figure 3. IMA demonstrated moderate discrimination for clinically suspected acute myocarditis (AUC 0.64, 95% CI 0.52–0.75). At the Youden-optimal cut-off (0.53), sensitivity was high (89.36%) but specificity was limited (38.30%). In contrast, conventional biomarkers particularly TnI and TnT showed higher discrimination, while CK-MB, NT-proBNP, and CRP also achieved good performance. Collectively, these results support positioning IMA as an adjunct/triage biomarker rather than a stand-alone diagnostic test. Consistent with this operating profile, IMA yielded a modest LR+ and a more informative LR−.

**Table 4:** Diagnostic performance of IMA and conventional biomarkers for discriminating clinically suspected acute myocarditis: ROC analysis.

Marker	AUC (95% CI)	Cut-off (Youden)	Sensitivity (%)	Specificity (%)	LR+	LR−
IMA (ABSU)	0.64 (0.52–0.75)	0.53	89.36	38.30	1.45	0.28
TnI (ng/mL)	0.96 (0.90–1.00)	0.11	91.49	100.00	∞	0.09
TnT (pg/mL)	0.93 (0.86–0.98)	6.86	85.11	97.87	39.96	0.15
CK-MB (ng/mL)	0.83 (0.73–0.91)	3.26	74.47	89.36	7.00	0.29
NT-proBNP (ng/L)	0.88 (0.81–0.94)	120.00	68.09	95.74	15.98	0.33
CRP (mg/L)	0.85 (0.76–0.92)	5.86	61.70	95.74	14.48	0.40

AUC, area under the curve; CI, confidence interval. Optimal cut-offs were derived using the Youden index. LR+ = sensitivity/(1−specificity); LR− = (1−sensitivity)/specificity. For markers with specificity = 100% at the selected cut-off, LR+ is not finite.

**Figure 4:**
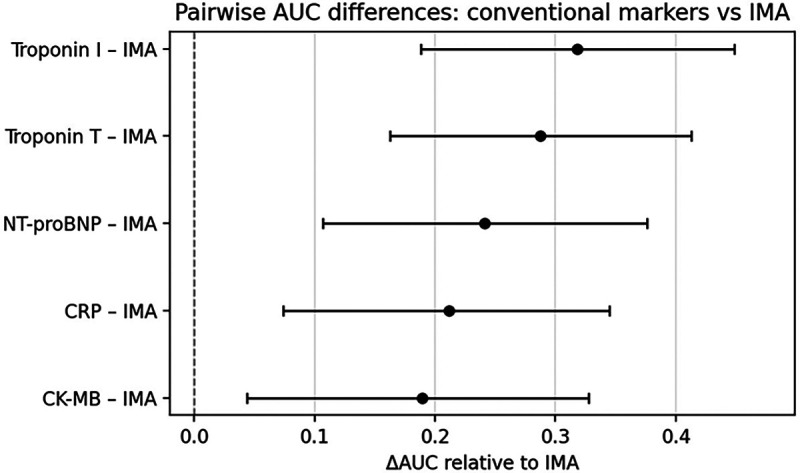
Pairwise differences in AUC between conventional biomarkers and IMA. Points represent pairwise bootstrap *Δ*AUC estimates (comparator minus IMA), and horizontal bars indicate 95% confidence intervals. Positive values indicate better discrimination than IMA.

Pairwise AUC comparisons further clarified this hierarchy and are illustrated in Figure 4. IMA lagged behind all conventional biomarkers, with the largest gaps observed vs. troponin I (ΔAUC 0.318, 95% CI 0.188–0.449), troponin T (ΔAUC 0.288, 95% CI 0.163–0.413), and NT-proBNP (ΔAUC 0.241, 95% CI 0.107–0.376). Even CK-MB and CRP outperformed IMA (ΔAUC 0.189, 95% CI 0.044–0.328 and 0.212, 95% CI 0.074–0.345, respectively), reinforcing that IMA ranked behind established markers in discriminatory performance.

Importantly, higher IMA values were not associated with the selected indicators of presentation severity available in our dataset, including arrhythmia/rhythm disturbance, echocardiographic abnormalities, left ventricular systolic dysfunction, pericardial effusion, or length of hospital stay. Although these exploratory analyses do not exclude prognostic utility, they do not support a clear role for IMA as a marker of short-term clinical severity in the present cohort. At the same time, the apparent superiority of conventional biomarkers over IMA should be interpreted with caution. Troponin I, troponin T, CK-MB, and NT-proBNP were part of the supportive criteria used to classify children as having clinically suspected myocarditis in this pragmatic study design. Their subsequent evaluation in ROC analyses therefore introduces a risk of incorporation bias, which may have inflated their AUCs relative to IMA. This issue does not negate the observed elevation of IMA in cases vs. controls, but it tempers direct claims about biomarker hierarchy.

### 3.2 Incremental discrimination

**Figure 5:**
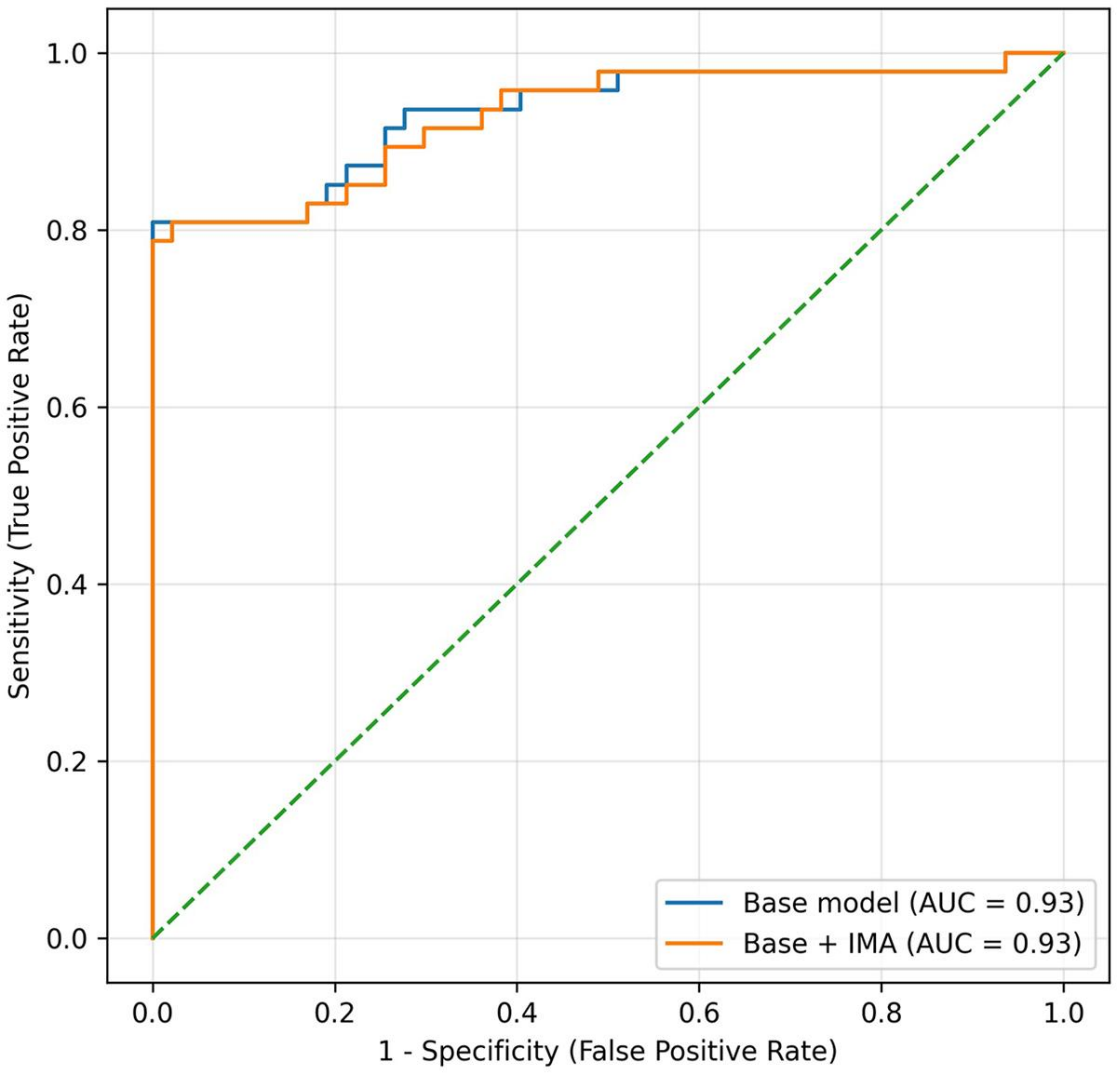
ROC curves for incremental models (base vs. Base + IMA). ROC curves were constructed from predicted probabilities of logistic regression models. The base model included log10-transformed CK-MB, NT-proBNP, and CRP; the extended model added IMA. Model AUCs and *Δ*AUC (with uncertainty and *p*-value) were evaluated by bootstrap resampling and are reported in the Results.

Incremental ROC curves for the base model vs. base + IMA are shown in Figure 5. Adding IMA increased AUC from 0.92 to 0.93 (ΔAUC 0.01; 95% CI: 0.00–0.03; *p* = 0.153). This minimal change indicates that IMA contributed little additional discrimination once conventional biomarkers were already incorporated into the model.

### 3.3 Correlation analysis

**Figure 6:**
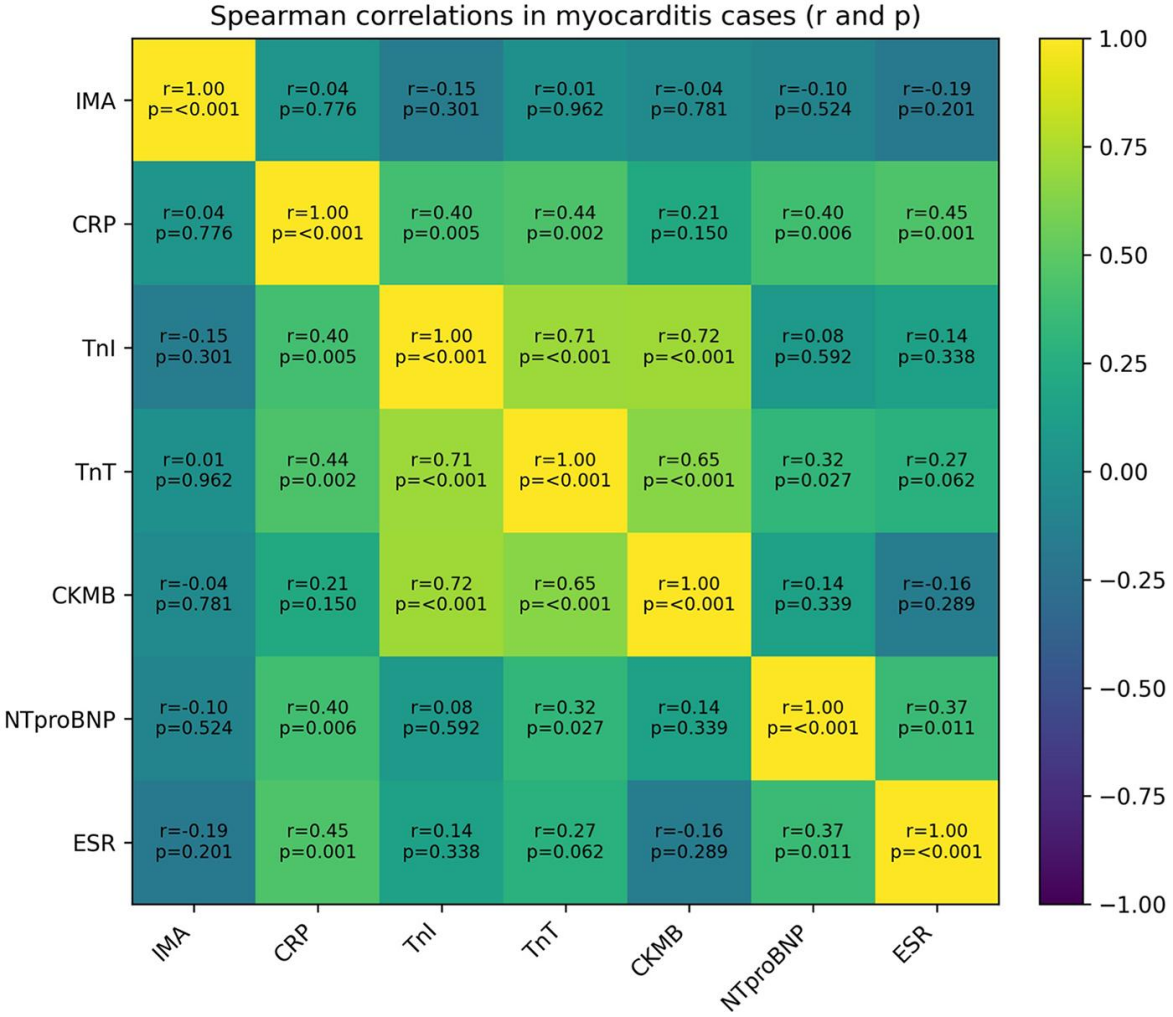
Spearman correlation heatmap in clinically suspected acute myocarditis cases (*r* and *p* values). Heatmap displays Spearman's *ρ* for pairwise associations among biomarkers within the clinically suspected acute myocarditis group; each cell reports *ρ* along with the corresponding *p*-value.

Spearman correlations among biomarkers in myocarditis cases are shown in Figure 6 (r and *p* values displayed). The strongest positive correlations were observed among conventional injury biomarkers, particularly between troponin I and CK-MB (*ρ* = 0.719), troponin I and troponin T (*ρ* = 0.706), and troponin T and CK-MB (*ρ* = 0.647). By contrast, IMA showed only weak correlations with the conventional biomarkers (all |*ρ*| ≤ 0.190), suggesting that it may reflect a partly distinct and less disease-specific biological signal.

**Figure 7:**
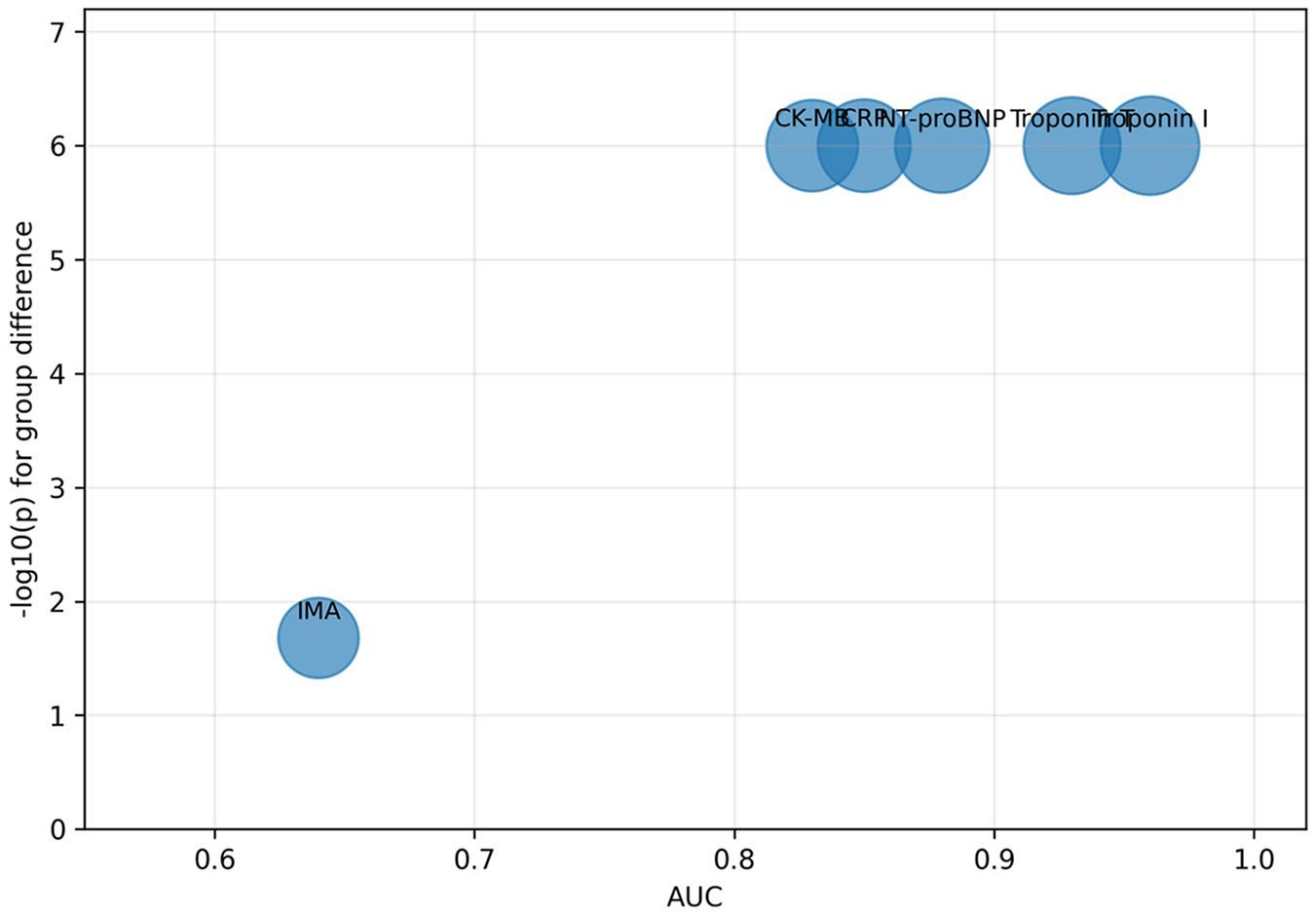
Bubble plot summarizing AUC and strength of group differences (−log10 p). The *x*-axis indicates AUC from ROC analysis (Table 4), and the *y*-axis indicates −log10**(p)** from between-group comparisons (Table 1). Bubble size is proportional to AUC. This plot provides a visual summary of diagnostic discrimination alongside statistical evidence of group separation and does not replace Tables 1 and 4.

A bubble plot summarizing discrimination (AUC) and strength of group differences is provided in Figure 7. As shown in Figure 7, IMA was positioned clearly behind the conventional biomarkers, reflecting both its lower discriminatory accuracy and its weaker between-group separation. In contrast, the conventional biomarkers clustered in the upper-right region of the plot, indicating both stronger group separation and better overall discriminatory performance.

## 4 Discussion

The present study evaluated ischemia-modified albumin (IMA) as a potential adjunctive biomarker in children with clinically suspected acute myocarditis, given the biological link between oxidative stress and myocardial inflammation (9). Our findings confirm that IMA levels are elevated in children with clinically suspected acute myocarditis compared with healthy peers, a pattern consistent with earlier reports in both pediatric and adult populations. In pediatric inflammatory conditions, elevated IMA levels have also been reported outside myocarditis. For example, in children with COVID-19, IMA levels at admission were significantly higher than in healthy controls, further supporting the view that IMA is responsive to oxidative and inflammatory stress but is not disease-specific (16). Similarly, a conference abstract from the COSMED V study by Bentancur et al. reported higher IMA levels in adults presenting with acute myocarditis and suggested an association with less favorable subsequent outcomes (14).

Despite this biological plausibility and supportive literature, the diagnostic performance of IMA in the present cohort was limited. As a standalone biomarker, IMA showed only modest discrimination. With an area under the ROC curve of 0.64, it correctly identified most cases (sensitivity 89%) but at the cost of many false positives (specificity only 38%). This pattern, characterized by high sensitivity with limited specificity has been observed across a range of pediatric conditions where IMA has been investigated. In pediatric conditions such as COVID-19, IMA has been investigated as a potential diagnostic adjunct, but its interpretation remains constrained by limited disease specificity and overlap with broader inflammatory and oxidative processes (16). In other pediatric inflammatory conditions, such as acute rheumatic fever, IMA similarly demonstrates elevated levels during the acute phase but lacks the specificity to distinguish cardiac involvement from systemic inflammation alone (13). Even in conditions as seemingly disparate as neonatal asphyxia and severe diarrhea, IMA has been proposed as an early marker of myocardial injury, reflecting its sensitivity to oxidative damage rather than any cardiac-specific process (9).

The elevation of IMA in clinically suspected acute myocarditis may be explained by its underlying biology rather than by disease-specific diagnostic performance. Under conditions of oxidative stress, which are abundant in inflamed heart muscle, the N-terminal end of albumin undergoes structural changes that reduce its ability to bind cobalt, the very change that the IMA test detects (9, 10). However, this process is not confined to the heart and also occurs in many other inflammatory and infectious states (13). This lack of tissue specificity is compounded by the fact that IMA is influenced by factors beyond myocardial injury, including alterations in serum albumin concentration and pH (9). Consequently, while IMA reliably signals the presence of oxidative stress, it struggles to distinguish myocarditis from the myriad other conditions that provoke a similar systemic inflammatory response.

Compared with conventional biomarkers, IMA demonstrated substantially weaker diagnostic discrimination. Troponin I, troponin T, and NT-proBNP all demonstrated excellent discrimination, with AUC values of 0.96, 0.93, and 0.88, respectively. These figures align with contemporary diagnostic standards in pediatric myocarditis, where multiparametric approaches consistently achieve high diagnostic accuracy. A recent study by Yuan and colleagues involving pediatric patients with acute myocarditis reported that NT-proBNP showed strong performance for identifying fulminant myocarditis, with an AUC of 0.935 at the reported cut-off (17). Similarly, in a cohort of 31 children with myocarditis, Price and colleagues found that cardiac troponin I had a sensitivity of 96.7% and ECG changes had a sensitivity of 96.8%, while cardiac magnetic resonance (CMR) achieved 100% sensitivity, underscoring the superiority of these established diagnostic tools (18). The 2018 revised Lake Louise Criteria for CMR, incorporating parametric mapping, have further enhanced diagnostic accuracy, with reported sensitivity and specificity exceeding 85% in pediatric populations (8, 19). This inferiority was not only visually apparent in the summary plots but was also confirmed quantitatively by pairwise AUC comparisons, in which IMA consistently underperformed troponin I, troponin T, NT-proBNP, CK-MB, and CRP (Figure 4).

CMR has become the key non-invasive imaging modality in the contemporary evaluation of pediatric myocarditis, while endomyocardial biopsy remains the historical reference standard and is reserved for selected cases (1, 2). Contemporary reviews, utilization studies, and survey data indicate that CMR use is increasing but remains heterogeneous across centers and healthcare systems (20–24). In a recent survey-based study, only 35% of centers reported routine CMR use in all children with suspected myocarditis, and although 72% reported using the revised Lake Louise criteria, scanner-specific pediatric normative mapping data were available in only 11% of centers for T1 mapping and 22% for T2 mapping (20). Consistent with this variability, a large multicenter Chinese cohort reported that cardiac magnetic resonance imaging was performed in only 4.9% of children with clinical myocarditis (24). In this context, inexpensive and readily available serum biomarkers remain attractive adjunctive tools, but they should be interpreted as complementary to established markers of myocardial injury and ventricular stress rather than as substitutes for imaging or conventional cardiac biomarkers (1, 21, 23).

From a pathobiological perspective, the modest standalone discrimination of ischemia-modified albumin (IMA) observed in our cohort is biologically plausible, because IMA is a nonspecific marker generated under conditions of ischemia, oxidative stress, and acidosis and is characterized by reduced transition-metal binding and limited tissue specificity (9, 25). This lack of specificity may explain why IMA was elevated in cases compared with healthy controls yet may provide less disease-specific information than biomarkers that more directly reflect myocardial injury or ventricular stress (1, 21). Prior literature has likewise suggested that the potential clinical role of IMA lies mainly in adjunctive, sensitivity-oriented assessment, particularly when interpreted alongside established tests, rather than as a stand-alone diagnostic marker (25–27). Accordingly, in pediatric myocarditis, IMA is better interpreted as a complementary biomarker to conventional cardiac markers and imaging findings, not as a substitute for them (1, 21, 26, 27).

The addition of IMA to a model already containing established biomarkers did not meaningfully improve diagnostic accuracy. The increase in AUC was negligible (*Δ*AUC 0.01, 95% CI −0.00 to 0.03). This finding is biologically and statistically plausible. The base model, which included CK-MB, NT-proBNP, and CRP, already captured complementary aspects of myocardial injury, hemodynamic stress, and systemic inflammation (1). The marginal improvement after adding IMA suggests that the oxidative stress signal captured by IMA largely overlaps with information already provided by these established markers. This does not diminish the biological relevance of IMA, but it does indicate that in a diagnostic panel already containing highly sensitive cardiac biomarkers, IMA contributes little additional discriminative information.

These findings do not exclude a potential adjunctive role for IMA in selected early or equivocal presentations. Its high sensitivity and low negative likelihood ratio suggest that this possibility warrants further investigation. However, our study did not include disease controls, serial sampling, symptom-to-sampling intervals, or prospective clinical decision outcomes. Accordingly, any proposed rule-out or triage role should be regarded as hypothesis-generating rather than practice-informing. Existing IMA literature supports, at most, an adjunctive and sensitivity-oriented role rather than replacement of conventional cardiac biomarkers (25–27).

Several limitations of our study should be acknowledged. First, the case–control design, while efficient for exploratory biomarker studies, does not fully reflect real-world diagnostic settings where the differential diagnosis includes viral infections and other inflammatory conditions. The absence of a disease-control group limits our ability to assess how well IMA distinguishes clinically suspected myocarditis from these entities. Second, the diagnosis of clinically suspected myocarditis was based on pragmatic clinical, laboratory, and imaging criteria rather than endomyocardial biopsy or uniform cardiac magnetic resonance confirmation. Third, because troponins, CK-MB, and NT-proBNP were part of the supportive case definition, ROC estimates for conventional biomarkers are susceptible to incorporation bias and may overstate their apparent discriminatory performance relative to IMA. Fourth, we used only admission samples; serial measurements and symptom-to-sampling intervals were unavailable. Fifth, although pre-analytical handling procedures were standardized and duplicate measurements were performed, formal study-specific intra-assay and inter-assay coefficients of variation and separate calibration or quality-control records were not available for retrospective reporting. Sixth, we did not adjust IMA for baseline albumin concentration, a factor known to influence results. Pre-analytical and biological variability related to albumin concentration and pH should therefore be considered when interpreting IMA measurements. Finally, without long-term follow-up, we cannot evaluate whether IMA might have prognostic value, an area that remains open for future investigation (1). Variables related to sequelae and unfavorable outcomes were not systematically available, limiting prognostic inference. Importantly, the present study was designed primarily to assess diagnostic discrimination rather than long-term prognostic performance. Relatedly, because symptom onset-to-sampling time was not recorded, we could not directly test whether IMA offers incremental rule-out value during an ultra-early presentation window.

Despite these limitations, our study has important strengths. We applied rigorous statistical methods, including bootstrap resampling for robust confidence intervals around AUC and *Δ*AUC. Diagnostic criteria followed the most recent AHA and ACC guidance (1, 2). We also reported likelihood ratios, which are more clinically interpretable than sensitivity and specificity alone. By matching controls for age and sex, we minimized demographic confounders. To our knowledge, this is the first study to systematically assess IMA's diagnostic performance and incremental value in children with clinically suspected acute myocarditis using such a comprehensive statistical framework, an approach that builds upon earlier descriptive studies in this population (9, 13).

Taken together, these findings indicate that IMA is elevated in children with clinically suspected acute myocarditis, but its diagnostic performance is modest and its incremental value beyond conventional biomarkers is limited. Any potential role as an adjunct in early or uncertain presentations should therefore be viewed as hypothesis-generating. For now, IMA cannot replace troponin or natriuretic peptides. Future prospective, multicenter studies that include disease-control groups, standardized sampling protocols, and longitudinal outcomes will be needed to determine whether IMA has a definitive role in the management of this condition (1). In the meantime, the combination of high-sensitivity troponin, natriuretic peptides, and advanced imaging, particularly CMR with parametric mapping, remains the diagnostic cornerstone for children with clinically suspected acute myocarditis (1, 2, 8, 19).

## 5 Conclusion

IMA is elevated in children with clinically suspected acute myocarditis but demonstrates modest standalone discrimination and limited incremental value beyond conventional biomarkers. Its most defensible role, if any, is as an adjunctive marker in clinically equivocal presentations. Any proposed triage role in very early presentations should be regarded as hypothesis-generating and requires prospective validation with serial sampling, disease-control groups, and imaging phenotyping.

## Data Availability

The raw data supporting the conclusions of this article will be made available by the authors, without undue reservation.
